# Brachioradial Pruritus Due to Cervical Spine Pathology

**DOI:** 10.2196/39863

**Published:** 2022-09-14

**Authors:** Maria Grabnar, Maneesh Tiwari, Jayesh Vallabh

**Affiliations:** 1 Ohio State University Columbus, OH United States

**Keywords:** spine, pruritus, cervical, spine, pathology, neck, dorsolateral, pain, weather, symptoms, patient, disease, trauma, neurological, exam, clinic, case study, magnetic resonance imaging, skin, itching, itchy, itchiness, dermatology, dermatologist, case report

## Introduction

Brachioradial pruritus is a skin condition that involves itching or pain most commonly involving the dorsolateral upper extremities. It is speculated that both cervical spine disease and sun-induced cutaneous nerve injury are important contributors, with varying degrees of presentation [1]. Patients often present with a history of sun exposure and are mostly middle-aged and female [2]. It has been postulated that neuropathic brachioradial pruritus may be the result of UV damage to nerve endings in an at-risk population with cervical spine pathology [3].

## Case

**Figure 1:**
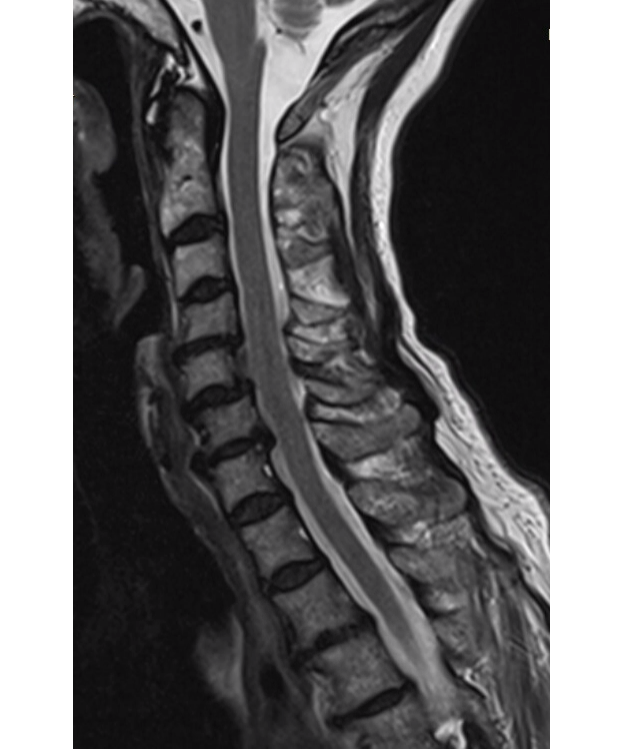
Midline sagittal view showing spondylosis that is worst at C5-C6 and C6-C7.

**Figure 2:**
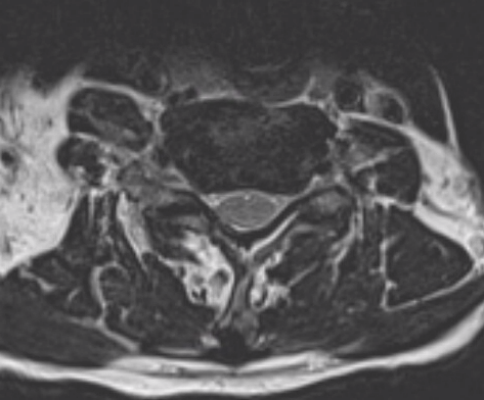
Axial view at C5-C6 showing significant neuroforaminal narrowing bilaterally.

A 70-year-old female patient with no past medical history presented to the outpatient spine clinic with a 2-year history of intermittent pruritus predominantly along the bilateral dorsolateral forearm. Symptoms were often severe enough that scratching resulted in open sores on her forearms. However, she denied any axial or radicular pain. She reported no known triggers except for flares occurring more frequently and with more severe symptomatology after sun exposure. The patient reported no prior dermatological diseases, familial pruritus, or trauma to the spine or extremities. She initially saw a dermatologist who deemed that the symptoms were not attributed to primary skin disease or inflammatory disorder. She then saw a rheumatologist who did not find any source of inflammatory disease. Magnetic resonance imaging of the C spine was obtained, and the patient was referred to a comprehensive spine center due to findings of bilateral neuroforaminal stenosis that was most severe at C4-C5 and C5-C6 (Figures 1 and 2). At the time of the spine clinic visit, she was asymptomatic; however, potential interventional options such as cervical epidural steroid injection were discussed in the event that her symptoms recurred. Prior to being evaluated in the spine clinic, she was taking prophylactic subsalicylate and loratadine due to mild alleviation of symptoms during a recent flare. The physical exam was unremarkable.

## Discussion

Itch is a complex neurologic phenomenon whose pathogenesis is only partially understood. It is speculated that irritation of spinal itch neurons caused by degenerative spinal changes leads to the spontaneous firing of damaged neurons, loss of the feedback mechanism for their descending inhibitory neurons, and loss of inhibitory interneurons that results in spinal hyperexcitability [3].

There are no established treatment guidelines for BPR. Various treatments have been described in the literature, each with mixed success. The conservative approach focuses on avoidance of sun exposure and neuropathic medications. A case series of 3 female patients with an average age of 66 years demonstrated complete resolution of symptoms in 2 of the 3 patients treated with computed tomography–guided cervical root nerve block at the levels of greatest stenosis [4]. A recent case report of a patient with brachioradial pruritus who underwent multilevel cervical diskectomy and fusion for cervical nerve root compression was found to be symptom-free afterward. Prior to surgery, he did receive temporary relief with epidural steroid injections [5].

This case is unique given that our patient presented with solely pruritus and without any history of pain related to her cervical spine pathology.
