# Correction: Brachioradial Pruritus Due to Cervical Spine Pathology

**DOI:** 10.2196/55896

**Published:** 2023-12-29

**Authors:** Maria Grabnar, Maneesh Tiwari, Jayesh Vallabh

**Affiliations:** 1 Ohio State University Columbus, OH United States

In “Brachioradial Pruritus Due to Cervical Spine Pathology” (JMIR Dermatol 2022;5(3):e39863) the authors noted one error.

The term “pruritis” was used instead of "pruritus" in several instances across the manuscript. This term was changed within the Keywords section, along with four other locations as follows:

The first sentence of the "Case" section was present as:

A 70-year-old female patient with no past medical history presented to the outpatient spine clinic with a 2-year history of intermittent pruritis predominantly along the bilateral dorsolateral forearm.

And will now appear as:

A 70-year-old female patient with no past medical history presented to the outpatient spine clinic with a 2-year history of intermittent pruritus predominantly along the bilateral dorsolateral forearm.

The fifth line under "Case" appeared as:

The patient reported no prior dermatological diseases, familial pruritis, or trauma to the spine or extremities.

And will now be changed to:

The patient reported no prior dermatological diseases, familial pruritus, or trauma to the spine or extremities.

The final line of the "Discussion" section previously appeared as:

This case is unique given that our patient presented with solely pruritis and without any history of pain related to her cervical spine pathology.

And will now be changed to:

This case is unique given that our patient presented with solely pruritus and without any history of pain related to her cervical spine pathology.

Finally, the title was presented as:

Brachioradial Pruritis Due to Cervical Spine Pathology

And will now appear as:

Brachioradial Pruritus Due to Cervical Spine Pathology

The correction will appear in the online version of the paper on the JMIR Publications website on December 29, 2023 together with the publication of this correction notice. Because this was made after submission to PubMed, PubMed Central, and other full-text repositories, the corrected article has also been resubmitted to those repositories.

